# Triglyceride-containing lipoprotein sub-fractions and risk of coronary heart disease and stroke: A prospective analysis in 11,560 adults

**DOI:** 10.1177/2047487319899621

**Published:** 2020-01-29

**Authors:** Roshni Joshi, S Goya Wannamethee, Jorgen Engmann, Tom Gaunt, Deborah A Lawlor, Jackie Price, Olia Papacosta, Tina Shah, Therese Tillin, Nishi Chaturvedi, Mika Kivimaki, Diana Kuh, Meena Kumari, Alun D Hughes, Juan P Casas, Steve Humphries, Aroon D Hingorani, A Floriaan Schmidt

**Affiliations:** 1 Institute of Cardiovascular Science, Faculty of Population Health, University College London, UK; 2 Department of Primary Care & Population Health, Faculty of Population Health, University College London, UK; 3 MRC Integrative Epidemiology Unit at the University of Bristol, UK; 4 Bristol NIHR Biomedical Research Centre, UK; 5 Population Health Science, Bristol Medical School, UK; 6 The Usher Institute of Population Health Sciences and Informatics, University of Edinburgh, UK; 7 Department of Epidemiology and Public Health, University College London, UK; 8 MRC Unit for Lifelong Health and Ageing, UK; 9 Institute for Social and Economic Research, University of Essex, UK; 10 Massachusetts Veterans Epidemiology Research and Information Center (MAVERIC), VA Boston Healthcare, USA; 11 Department of Cardiology, Division Heart and Lungs, University Medical Centre Utrecht, The Netherlands

**Keywords:** Epidemiology, triglycerides, lipoproteins, metabolomics, coronary heart disease, stroke

## Abstract

**Aims:**

Elevated low-density lipoprotein cholesterol (LDL-C) is a risk factor for cardiovascular disease; however, there is uncertainty about the role of total triglycerides and the individual triglyceride-containing lipoprotein sub-fractions. We measured 14 triglyceride-containing lipoprotein sub-fractions using nuclear magnetic resonance and examined associations with coronary heart disease and stroke.

**Methods:**

Triglyceride-containing sub-fraction measures were available in 11,560 participants from the three UK cohorts free of coronary heart disease and stroke at baseline. Multivariable logistic regression was used to estimate the association of each sub-fraction with coronary heart disease and stroke expressed as the odds ratio per standard deviation increment in the corresponding measure.

**Results:**

The 14 triglyceride-containing sub-fractions were positively correlated with one another and with total triglycerides, and inversely correlated with high-density lipoprotein cholesterol (HDL-C). Thirteen sub-fractions were positively associated with coronary heart disease (odds ratio in the range 1.12 to 1.22), with the effect estimates for coronary heart disease being comparable in subgroup analysis of participants with and without type 2 diabetes, and were attenuated after adjustment for HDL-C and LDL-C. There was no evidence for a clear association of any triglyceride lipoprotein sub-fraction with stroke.

**Conclusions:**

Triglyceride sub-fractions are associated with increased risk of coronary heart disease but not stroke, with attenuation of effects on adjustment for HDL-C and LDL-C.

## Introduction

Elevated low-density lipoprotein cholesterol (LDL-C) is thought to play a central role in atherogenesis^1^ and is associated with increased risk of coronary heart disease (CHD) in observational studies, an association which is robust to adjustment for other risk factors.^2^ Randomised controlled trials of LDL-C lowering drugs also now provide compelling evidence of its causal role in CHD.^3^ On the other hand, the role of triglycerides in CHD is less clear. Observational data from a large meta-analysis of prospective studies also suggest a higher circulating concentration of triglycerides, and a lower concentration of high-density lipoprotein cholesterol (HDL-C) is associated with CHD but the association of each is attenuated to the null after adjustment for the other,^2^ leading to uncertainty on the nature of these associations with CHD. However, recently, Mendelian randomisation studies have suggested a potential causal association between triglycerides and CHD,^4^ which has gained some support following publication of the findings of the REDUCE-IT trial.^5^

Total circulating triglyceride concentration is made up of contributions from a number of different triglyceride-containing lipoprotein sub-fractions which, as yet, are not routinely measured in clinical practice. Triglycerides are most abundant in chylomicrons transporting fatty acids from the intestine after a meal, and very low-density lipoproteins (VLDLs), transporting triglycerides from the liver.^6^ In general, the concentration of triglycerides decreases as the lipid content of these lipoproteins is hydrolysed and, thus, different lipoprotein sub-fractions may display different associations with CHD risk.^7^

Recently, a high throughput technology was developed that enables the quantification of triglyceride-containing lipoprotein sub-fractions, among other lipoprotein subclasses and other metabolites, using serum nuclear magnetic resonance (NMR).^8^ A study based on this platform in a prospective cohort in China found evidence that the association of triglycerides with CHD may depend on the type of triglyceride-containing lipoprotein sub-fraction.^9^ A further study used the same platform in the Finnish population.^10^ However, no study to our knowledge has yet investigated the association of triglyceride-containing lipoprotein sub-fractions with risk of CHD or stroke in other populations. The current paper describes an observational analysis of 14 triglyceride-containing sub-fraction measurements in 11,560 participants to investigate potential sub-fraction specific associations with CHD and stroke in prospective longitudinal cohort studies from the University College London (UCL)–Edinburgh–Bristol (UCLEB) Consortium.^11^

## Methods

### Study samples

The design and data collection for the UCLEB Consortium of longitudinal population studies has been described previously.^11^ NMR metabolite measurements for the current analysis were available in 11,560 participants enrolled in the British Regional Heart Study,^12^ including men aged 60–79 years at metabolite assessment in 1998–2000 and seven years of follow-up; the Whitehall II study,^11^ including UK government workers aged 45 to 69 years at metabolite assessment in 1997–1999 and seven years of follow-up; and The Southall And Brent REvisited Study^13^ (SABRE), a tri-ethnic study including British men and women of European (SABRE1), South Asian (SABRE2) and African-Caribbean descent (SABRE3), with 20 years of follow-up.

### Metabolite quantification

The Nightingale high-throughput NMR metabolomics platform was used to quantify concentrations of total and 14 triglyceride-containing lipoprotein sub-fraction metabolomics measures (referred to as triglyceride sub-fractions from here onwards) from serum samples in either fasting or non-fasting states in all contributing studies. Apolipoprotein A1 (apoA1) and B (apoB) were measured using the same platform. Detailed experimental protocols and application of the metabolomics platform method have been described and reviewed elsewhere.^8,14,15^

### Participant characteristics

The following participant information was recorded at time of metabolite measurement: age (years), sex, lifestyle factors; smoking (categorised here as ever/never) and alcohol (ever/never); body mass index (BMI; kg/m^2^); and systolic and diastolic blood pressure (mmHg) and type 2 diabetes mellitus (yes/no). Standard clinical chemistry was used to measure LDL-C and HDL-C (mmol/L) and serum triglycerides (mmol/L). This included, in all three studies, LDL-C levels being estimated using Friedwald’s equation from total cholesterol and triglycerides (LDL-C = total cholesterol – (triglyceride/5) – HDL).^16^

### Outcomes

Incident CHD was defined as the first occurrence of fatal or non-fatal myocardial infarction (MI), or coronary revascularisation. Incident stroke was defined as the first occurrence of fatal or non-fatal ischaemic or haemorrhagic stroke. Methods of disease ascertainment for contributing UCLEB cohorts are described in detail elsewhere.^11^

### Statistical analysis

**Figure 1.**
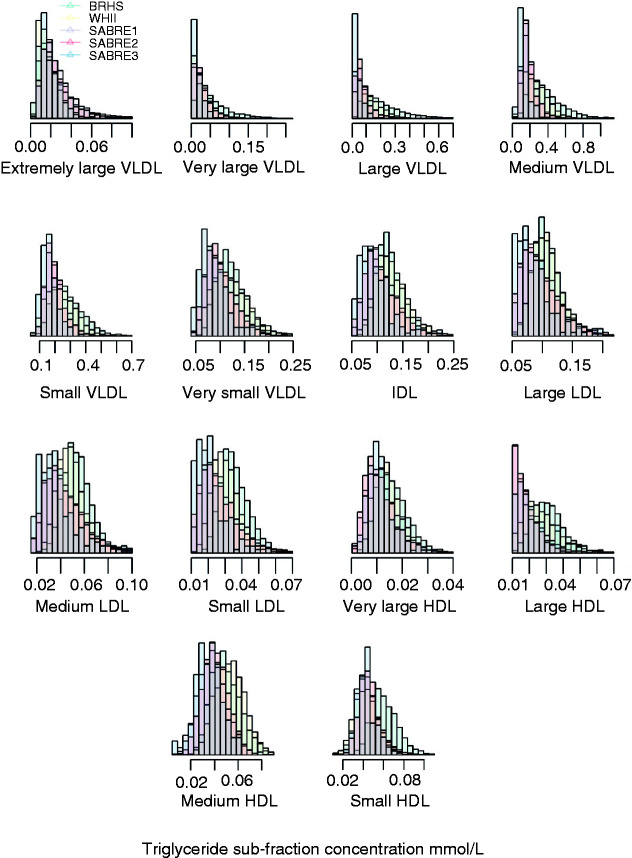
Distributions of 14 triglyceride sub-fractions. Histograms show study specific distribution (mmol/L) of each nuclear magnetic resonance triglyceride sub-fraction. BRHS: British Regional Heart Study; WHII: Whitehall II study; SABRE: The Southall And Brent REvisited Study; VLDL: very low-density lipoprotein; IDL: intermediate-density lipoprotein; HDL: high-density lipoprotein.

We first assessed the study-specific distribution of each triglyceride sub-fraction (Figure 1). On finding agreement across studies, sub-fraction measurements were mean centred and standardised to an SD of 1. Spearman’s correlation coefficient (*r*_s_) was used to explore associations between the 14 triglyceride sub-fractions, NMR-measured total serum triglyceride, and various participant characteristics. Study-specific logistic regression was used to estimate the odds ratio (OR) and 95% confidence interval (CI) with CHD and stroke. Estimates were synthesised across cohorts, using the fixed-effect inverse variance weighted estimator. We evaluated different multivariable logistic regression models to assess how much of the association with CHD and stroke remained after accounting for known CVD risk factors, specifically; age and sex (model 1), model 1 with additional correction for BMI, smoking, systolic blood pressure (SBP) and type 2 diabetes (model 2). For comparisons with previous meta-analysis^2^ and to assess independent association of the 14 sub-fraction associations with CHD, we subsequently conditioned on LDL-C and HDL-C measurements (model 3) or apoA1 and apoB (model 4). Given the potential influence of food intake on the concentration of circulating triglyceride sub-fractions, we compared the association of fasted and non-fasted triglyceride sub-fractions with CHD and stroke using data from the SABRE study. There is a reported interaction of diabetes and triglycerides;^17^ we performed a type 2 diabetes stratified analysis to determine whether such an interaction was present for the 14 triglyceride sub-fractions and CHD.

Analysis was conducted using R studio version 1.1.423^18^ using the following packages: visualise the correlation matrix (Corrplot),^19^ conduct meta-analyses (Metafor).^20^

## Results

The association of the 14 triglyceride sub-fractions and total NMR-measured triglycerides with CHD and stroke was assessed in a sample of 11,560 participants, of which 1031 experienced CHD and 582 stroke. The mean age was 58 (SD: 9.2) years, 7634 (66%) were men, mean BMI was 26.4 (SD: 3.8), and average SBP was 131 (SD: 22.7) mmHg; see Table 1.

**Table 1.** Description of study populations.

	SABRE2 *N* = 1526	SABRE1 *N* = 1561	SABRE3 *N* = 192	WHII *N* = 4728	BRHS *N* = 3553	Total participant sample *N* = 11,560	Missing %
Age, years	51.1 (7.0)	53.2 (7.3)	53.3 (5.7)	55.5 (6.0)	68.7 (5.5)	58.6 (9.2)	0.00
Sex, male (%)	1278 (83.8)	1356 (86.9)	180 (93.8)	1267 (26.8)	3553 (100)	7634 (66.0)	0.00
BMI, kg/m^2^	26.1 (3.6)	26.1 (3.9)	26.8 (3.7)	26.1 (3.9)	26.9 (3.6)	26.4 (3.8)	0.06
Smoking, ever	349 (22.9)	1097 (70.3)	79 (41.4)	617 (14.6)	2521 (71.1)	4663 (42.2)	0.04
Alcohol, ever	899 (59.3)	1467 (94.0)	176 (92.2)	4244 (91)	3145 (90.4)	9931 (87.0)	0.01
Type 2 diabetes, (%)	318 (20.1)	86 (5.5)	37 (19.3)	251 (5.3)	379 (11.1)	1071 (9.4)	0.01
SBP, mmHg	125.2 (18.2)	123.0 (17.1)	128.4 (18.7)	122.9 (16.1)	149.2 (24.2)	131.3 (22.7)	0.00
DBP, mmHg	80.1 (10.6)	76.8 (10.6)	81.5 (12.4)	77.4 (10.3)	85.3 (11.2)	80.2 (11.3)	0.00
HbA1c, mmol/L	6.2 (1.3)	5.6 (0.6)	6.0 (0.8)	–	5.0 (0.9)	5.4 (1.0)	0.46
CRP, mg/L				2.0 (3.7)	3.4 (6.5)	2.6 (5.2)	0.29
IL-6, pg/mL				1.8 (1.5)	3.2 (3.0)	2.4 (2.4)	0.29
Clinical chemistry measured lipids				
Total cholesterol, mmol/L^a^	5.9 (5.2, 6.6)	6.1 (5.4, 6.8)	5.8 (5.1, 6.6)	5.8 (5.2, 6.6)	6.0 (5.4, 6.7)	5.9 (5.3, 6.6)	0.00
LDL-C, mmol/L^a^	3.8 (3.2, 4.4)	4.0 (3.4, 4.7	3.8 (3.2, 4.5)	3.8 (3.2, 4.4)	3.9 (3.3, 4.5)	3.8 (3.3, 4.5)	0.00
HDL-C, mmol/L^a^	1.2 (1.0, 1.4)	1.3 (1.1, 1.5)	1.4 (1.1, 1.6)	1.4 (1.2, 1.7)	1.3 (1.1, 1.5)	1.3 (1.1, 1.6)	0.06
TG, mmol/L^a^	1.7 (1.2, 2.5)	1.4 (1.0, 2.1)	1.1 (0.8, 1.5)	1.1 (0.8, 1.6)	1.6 (1.2, 2.2)	1.4 (1.0, 2.0)	0.05
NMR-measured lipids							
Total cholesterol, mmol/L^a^	3.8 (3.3, 4.4)	4.1 (3.5, 4.6)	3.8 (3.2, 4.4)	5.1 (4.5, 5.6)	4.6 (4.0, 5.2)	4.6 (3.9, 5.3	0.00
TG, mmol/L^a^	0.9 (0.7, 1.2)	0.9 (0.7, 1.2)	0.8 (0.6, 0.9)	1.2 (0.9, 1.5)	1.4 (1.1, 1.9)	1.2 (0.9, 1.5)	0.00
Apolipoprotein A1, mmol/L^a^	1.2 (1.1, 1.3)	1.2 (1.1, 1.3)	1.2 (1.1, 1.3)	1.6 (1.5, 1.7)	1.4 (1.3, 1.5)	1.4 (1.3, 1.6)	0.00
Apolipoprotein B, mmol/L^a^	0.8 (0.7, 0.9)	0.8 (0.7, 1.0)	0.8 (0.7, 0.9)	1.0 (0.9, 1.1)	1.0 (0.8, 1.1)	0.9 (0.8, 1.1)	0.00
CHD, events/*n*	317/919 (17.4)	189/1035 (15.4)	23/109 (17.4)	122/4200 (2.8)	380/3173 (10.7)	1031/10,467 (9.9)	0.09
Stroke, events/*n*	136/1191 (10.2)	119/1212 (8.9)	10/118 (13.2)	68/4646 (1.4)	241/3312 (6.8)	582/10,479 (5.3)	0.04

Values are mean ± SD, or %.

a Median (interquartile range).

SABRE: The Southall And Brent REvisited Study; WHII: the Whitehall II study; BRHS: British Regional Heart Study; BMI: body mass index; SBP: systolic blood pressure; DBP: diastolic blood pressure; LDL-C: low-density lipoprotein cholesterol; HDL-C: high-density lipoprotein cholesterol; TG: triglycerides; CRP: C-reactive protein; IL-6: interleukin-6; CHD: coronary heart disease.

**Figure 2.**
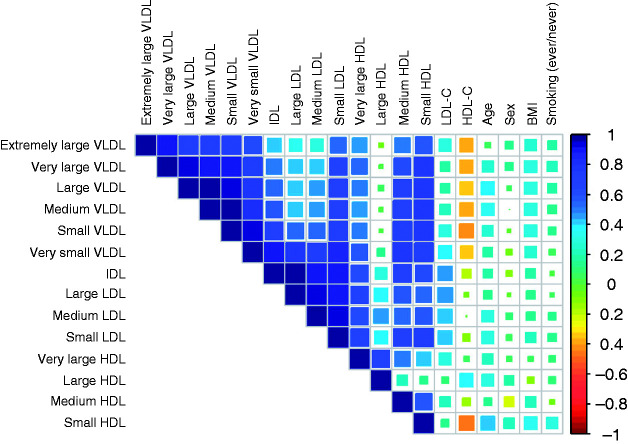
Correlation matrix heat map of 14 triglyceride sub-fractions and study variables. Heat map matrix represents Spearman's correlations. VLDL: very low-density lipoprotein; IDL: intermediate-density lipoprotein; LDL: low-density lipoprotein; HDL: high-density lipoprotein; LDL-C: LDL cholesterol; HDL-C: HDL cholesterol; BMI: body mass index.

The triglyceride sub-fractions showed positive correlations with one another (Figure 2), and clinical chemistry-measured lipid measurements. For example, triglycerides in extremely large VLDL was positively correlated with 12 sub-fractions with *r*_s_ in the range of 0.33 to 0.91. Various participant characteristics were correlated with triglyceride sub-fractions (*r*_s_ range −0.01 to 0.32), specifically BMI was positively correlated 14 triglyceride sub-fractions (*r*_s_ in the range 0.10 to 0.32). With the exception of triglycerides in large HDL, the remaining triglyceride sub-fractions (mainly in the VLDL subclass) exhibited a negative correlation with HDL-C (*r*_s_ range −0.42 to −0.33).

### Associations of triglyceride sub-fractions with CHD and stroke

**Figure 3.**
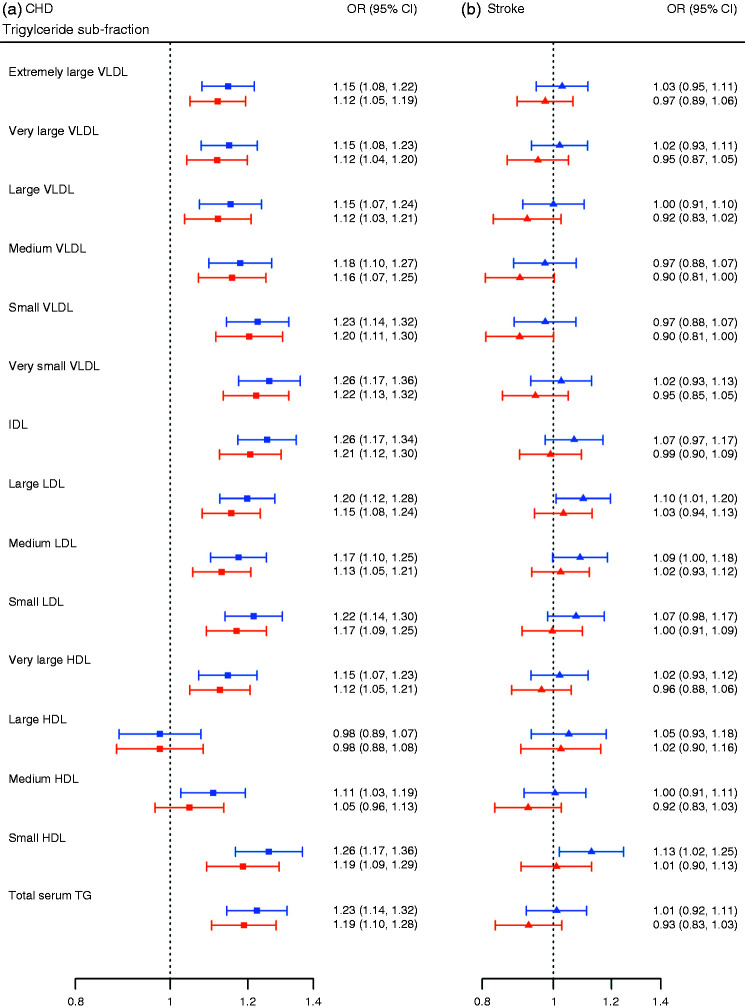
Nuclear magnetic resonance-measured total and 14 triglyceride sub-fraction associations with coronary heart disease and stroke. Effect estimates are presented as odds ratios with 95% confidence intervals per one standard deviation increase in the analyte for coronary heart disease (a) and stroke (b). Models are adjusted for: age and sex (model 1, denoted by blue bar), model 1 with additional correction for smoking status, body mass index, systolic blood pressure and type 2 diabetes (model 2, denoted by orange bar). CHD: coronary heart disease; OR: odds ratio; CI: confidence interval; VLDL: very low-density lipoprotein; IDL: intermediate-density lipoprotein; LDL: low-density lipoprotein; HDL: high-density lipoprotein; TG: triglyceride.

In age and sex adjusted analysis total triglycerides and all triglyceride sub-fractions, with the exception of triglycerides in large HDL, were associated with an increased risk of CHD (Figure 3). There was low to moderate (*I*^2 ^< 50%) heterogeneity for 13 sub-fraction estimates with CHD and stroke (Supplementary Material Table 1 online) and high heterogeneity (*I*^2 ^> 70%) in estimates for the large HDL sub-fraction. After additional adjustments for BMI, SBP, smoking and type 2 diabetes (model 2), effect estimates attenuated towards the null, with ORs in the range of 0.98 to 1.22. Triglycerides in the small and medium HDL sub-class were associated with an increased risk of CHD, whereas triglycerides in large HDL were inversely associated. Medium and large HDL sub-fractions did not exclude a neutral-effect. We determined to what extent LDL-C and HDL-C explained the independent association of triglyceride sub-fraction with CHD. Compared with model 2, additionally conditioning on LDL-C and HDL-C attenuated CHD ORs, with none of the 14 estimates showing convincing evidence of an independent effect (Supplementary Figure 1). We further explored the influence of replacing LDL-C and HDL-C with apoB and apoA1, which are considered more precise measurements. However, adjustment for apoA1 and apoB, rather than for HDL-C and LDL-C respectively, did not meaningfully change the results (Supplementary Figure 2), with one possible exception of triglycerides in the VLDL subclass for which the direction of associations changed. For example, accounting for LDL-C and HDL-C, ORs were in the range of 1.01 to 1.04, whereas adjustment of apoA1 and apoB yielded negative associations in the range of 0.96 to 0.99.

Associations of total and 14 triglyceride sub-fractions with stroke were smaller than those observed for CHD (Figure 3), with considerable attenuation in both direction and magnitude, after adjustments for SBP, BMI, smoking and type 2 diabetes (model 2) none were statistically significant.

Effect estimates of 14 triglyceride sub-fractions with CHD were comparable in direction and pattern of association using triglyceride measures quantified in the fasting and non-fasting state (Supplementary Figure 3).

The effect estimates of total serum triglycerides with CHD measured using NMR methods versus clinical chemistry methods were comparable in direction and magnitude of effect (NMR measured total triglyceride: OR 1.19, 95% CI 1.10 to 1.28, versus clinical chemistry measured total triglyceride: OR 1.11, 95% CI 1.07 to 1.21 (Supplementary Table 2).

### CHD associations in participants with and without type 2 diabetes

Given associations of triglyceride sub-fractions with CHD, we explored evidence of differences in effect in a type 2 diabetes (T2DM) stratified analysis (1071 T2DM participants *vs*. 10,347 non-T2DM participants). We did not find evidence of an interaction between triglyceride sub-fractions and diabetes with CHD risk (Table 2). Triglyceride concentration in 13 sub-fractions was positively associated with CHD in participants with and without T2DM, although effect estimates for the non-T2DM subgroup had wide 95% CIs that frequently included unity due to the comparatively small sample size.

**Table 2.** Effect estimates and upper and lower 95% confidence interval in subgroup participant population of type 2 diabetes and risk of coronary heart disease.

	Type 2 diabetes *N* = 1071	No type 2 diabetes *N* = 10,347	*p* value for interaction
	CHD OR (95% CI)	CHD OR (95% CI)	
Extremely large VLDL	1.05 (0.92, 1.20)	1.14 (1.06, 1.22)	0.286
Very large VLDL	1.04 (0.91, 1.18)	1.13 (1.04, 1.22)	0.262
Large VLDL	1.02 (0.88, 1.18)	1.13 (1.04, 1.23)	0.201
Medium VLDL	1.05 (0.91, 1.22)	1.17 (1.08, 1.28)	0.168
Small VLDL	1.11 (0.94, 1.30)	1.22 (1.13, 1.33)	0.261
Very small VLDL	1.15 (0.96, 1.36)	1.25 (1.15, 1.36)	0.364
IDL	1.22 (1.04, 1.43)	1.21 (1.12, 1.31)	0.903
Large LDL	1.19 (1.02, 1.39)	1.15 (1.06, 1.24)	0.667
Medium LDL	1.11 (0.95, 1.29)	1.13 (1.05, 1.22)	0.812
Small LDL	1.16 (1.00, 1.36)	1.17 (1.08, 1.26)	0.954
Very large HDL	1.08 (0.92, 1.25)	1.11 (1.03, 1.20)	0.685
Large HDL	0.91 (0.73, 1.14)	0.94 (0.85, 1.04)	0.814
Medium HDL	0.94 (0.78, 1.13)	1.05 (0.96, 1.14)	0.264
Small HDL	1.12 (0.94, 1.33)	1.21 (1.11, 1.33)	0.380

Estimates are odds ratios and (upper and lower bands). Odds ratios are adjusted for: age, sex, body mass index, smoking status, alcohol, systolic blood pressure.

CHD: coronary heart disease; OR: odds ratio; CI: confidence interval; VLDL: very-low-density lipoprotein; IDL: intermediate-density lipoprotein; LDL: low-density lipoprotein; HDL: high-density lipoprotein.

## Discussion

We found that total triglyceride and most triglyceride sub-fractions (with the exception of triglyceride in the HDL subclass) were associated with an increased risk of CHD. These associations persisted after accounting for differences in age, sex, SBP, BMI, smoking and T2DM, ranging from a CHD OR of 1.12 to 1.22. Importantly, triglyceride within the VLDL subclass had the strongest association with CHD (OR in the range of 1.12 to 1.22), which is consistent with the view that VLDL particles are particularly atherogenic.^21,22^ Accounting for HDL-C and LDL-C attenuated the CHD associations towards the null, suggesting that triglyceride associations are not independent of LDL-C and HDL-C. While such analyses may suggest an absence of a direct pathway, that is, independent of LDL-C and HDL-C, an alternative explanation for this attenuation may be found in the different variability of triglyceride measurements compared with HDL-C.^23^ Biological variability in triglyceride measurements, the extent of which we were not able to assess here, has previously been reported with a median variation of 23.5% and is a consideration when evaluating the role of triglycerides in CVD risk.^22^ We did not observe any interaction between T2DM status and the sub-fraction associations with CHD.

Consistent with a previous study,^9^ concentrations of triglycerides within large and medium HDL particles did not show an association with risk of CHD. Measures in the HDL sub-fraction likely represents the role of HDL particles that mediates reverse cholesterol transport, a different process from that indexed by the other sub-fractions. In relation to reverse cholesterol transport, variants in the cholesteryl ester transfer protein (CETP) gene and treatment with a potent, target-specific CETP inhibitor for a sufficient duration, both result in reduced triglycerides, LDL-C and apoB, elevated HDL-C and apoA1 and reduced CHD risk.^24^

The apparent inconsistency of associations of total triglycerides and CHD^2^ and uncertainty about causal relationships may be due in part to the complexity of triglyceride absorption and metabolism, and a potentially heterogeneous role of different triglyceride-containing lipoproteins in atherogenesis. Conventional measures of total serum triglyceride levels do not take into account the differing lipoprotein compositions with the same detailed precision as NMR technology and, thus, standard clinical chemistry measurement processes may not sufficiently delineate the relationship of individual lipoproteins with CHD. To evaluate this, we assessed the association of total serum triglycerides measured using clinical chemistry methods and NMR methods and report comparable effects despite the absolute difference in total serum triglyceride concentration between the two methods.

Triglycerides are most abundant in chylomicrons transporting fatty acids from the intestine, and VLDLs, transporting triglycerides from the liver.^6^ In general, the triglyceride content of lipoprotein sub-fractions decreases as the lipid pools of these lipoproteins are hydrolysed and, thus, different lipoproteins may be associated with differential CHD risk.^7^ Increases in total triglycerides may reflect an increase in the triglyceride content of lipoproteins, or an increase in the total number of triglyceride-rich (predominantly VLDL) particles. In addition, an emerging view is that the remnant cholesterol content (that can be proxied by total cholesterol – HDL-C – LDL-C) in triglyceride-rich lipoprotein in addition to triglycerides, has pro-atherogenic actions.^6,25,26^ A suggested mechanism is that triglycerides can penetrate the arterial intima and trigger inflammation, but are then metabolised, whereas the cholesterol remnants are not, promoting foam cell formation, atherosclerotic plaques and ultimately CHD.^27^

It has been suggested that apoB (which increases the total number of atherogenic particles) is more robustly associated with CHD than other measures.^28^ However, we observed associations that were comparable in direction and magnitude for CHD, regardless of whether adjustments were for LDL-C and HDL-C or apoA1 or apoB, with the possible exception of triglyceride in the VLDL subclass.

In contrast to associations with CHD, we found no convincing evidence of an association between triglyceride sub-fractions and risk of stroke. The magnitude of unadjusted and adjusted associations for stroke were all weaker than for CHD and also showed greater variation in direction between the triglyceride sub-fractions. However, it is possible that large studies with more stroke events might find evidence of a modest association of certain sub-fractions with stroke risk.

We compare the results presented here to existing knowledge in this area. A study using Finnish participant data to examine the association of circulating metabolites and cardiovascular disease (CVD) reported age and sex adjusted associations of triglycerides and incident CVD^10^ (800 cases/7,256 participants; hazard ratio: 1.25, 95% CI: 1.13 to 1.35). Similar to the findings presented here, Wurtz et al. report that triglyceride associations with CHD attenuate towards the null when performing additional adjustment for HDL-C. A recent study in participants from a Chinese cohort (912 cases/4662 participants) demonstrated consistent associations of triglyceride sub-fractions and incident MI after controlling for age, sex, fasting hours, region, smoking status and educational attainment (OR per 1 SD increase in the metabolite in the range 1.03–1.31);^9^ however, effect estimates attenuated towards the null with additional adjustment for SBP and BMI. The present study, although not controlling for fasting hours or educational attainment, identified comparable effect estimates which remained robust to adjustments for SBP and BMI.

This study has several strengths. We observed no large variation in the distribution of the 14 triglyceride sub-fractions when combining study specific distributions, indicating homogeneity in the spread of the 14 triglyceride sub-fractions and no discernible differences in the contributing populations from which the samples were derived. We include a tri-ethnic cohort and report a similar distribution of each analyte irrespective of ethnic group. Our findings were consistent with those reported by Holmes et al.^9^ and Wurtz et al.^10^ but included a larger sample size for CHD (CHD 1031 cases/10,467 controls, stroke 582 cases/10,479 controls) compared with Holmes et al. *(*MI 912 cases/1466 controls and ischaemic stroke 1146 cases/1466 controls) and Wurtz et al. (CVD 800 cases/7256 controls), thereby increasing precision in the effect estimates we present here. We also extend the investigation of the relationship between triglyceride-containing lipoprotein sub-fractions beyond ethnically distinct Finnish and Chinese populations. Here we demonstrate consistent findings using data from multiple cohorts across multiple ethnicities consisting of European, South-Asian and African-Caribbean descent, and across differing age groups.

### Study limitations

Some limitations deserve consideration. Total triglycerides and triglyceride-containing lipoprotein sub-fraction concentration in plasma samples are affected by food intake.^6^ Despite this, in a stratified analysis, we determined similar associations of triglyceride-containing lipoprotein subfractions with CHD and stroke among fasted and non-fasted subjects from the SABRE study.

In this study stroke was defined as a composite of ischaemic and haemorrhagic stroke. Lower triglyceride concentrations have been shown to be associated with decreased risk of haemorrhagic stroke, whereas higher triglyceride associated with increased risk of ischaemic stroke.^29^ We were unable to differentiate types of stroke in this study (in which typically there is a 4:1 ratio of ischaemic to haemorrhagic stroke events); as such the null effect estimates presented here may not reflect the true association of triglycerides and ischaemic stroke.

In addition, we were unable to account for lipid lowering medication or socio-economic position as has been conducted in previous work^10^ and may modify the effect of triglyceride sub-fraction associations with CHD, therefore it is possible that observed associations may be explained by residual confounding due to factors not included in this study.

## Conclusions

In conclusion, we demonstrate risk-increasing associations of 13 triglyceride sub-fractions with CHD, with varying effect estimates between sub-fractions, the strongest being for the triglycerides in the VLDL sub-fraction. By contrast, we did not observe similar associations for stroke. Further studies, for example using Mendelian randomisation, could assess which (if any) of the associations is causal, and if any causal associations might be modifiable by new or existing drugs.

## Supplemental Material

CPR899621 Supplemental material - Supplemental material for Triglyceride-containing lipoprotein sub-fractions and risk of coronary heart disease and stroke: A prospective analysis in 11,560 adults Click here for additional data file. Supplemental material, CPR899621 Supplemental material for Triglyceride-containing lipoprotein sub-fractions and risk of coronary heart disease and stroke: A prospective analysis in 11,560 adults by Roshni Joshi, S Goya Wannamethee, Jorgen Engmann, Tom Gaunt, Deborah A Lawlor, Jackie Price, Olia Papacosta, Tina Shah, Therese Tillin, Nishi Chaturvedi, Mika Kivimaki, Diana Kuh, Meena Kumari, Alun D Hughes, Juan P Casas, Steve Humphries, Aroon D Hingorani and A Floriaan Schmidt in European Journal of Preventive Cardiology
