# Supplementary material for: Triglyceride-containing lipoprotein sub-fractions and risk of coronary heart disease and stroke: A prospective analysis in 11,560 adults
Source: Eur J Prev Cardiol. 2020 Jan 29;27(15):1617–26. doi: 10.1177/2047487319899621 (PMC7707881; doi:10.1177/2047487319899621)
Supplement: CPR899621 Supplemental material - Supplemental material for Triglyceride-containing lipoprotein sub-fractions and risk of coronary heart disease and stroke: A prospective analysis in 11,560 adults [file CPR899621_Supplemental_material.pdf]

Online supplementary table and figures

Supplementary table 1, I^2 statistics for CHD and stroke

Supplementary table 2, Effect estimates for NMR measured and clinical chemistry measured lipids with CHD

Supplementary figure 1, Evaluate the association of NMR measured total and 14 triglyceride sub-fraction with CHD independent of HDL-C and LDL-C

Supplementary figure 2, NMR measured total and 14 triglyceride sub-fraction associations with CHD

Supplementary figure 3, Total and 14 triglyceride sub-fraction measured in the fasting state and non-fasting state associations with CHD and stroke

Supplementary table 1 I^2 statistics for CHD and stroke

	CHD I^2 [95% CI]	Stroke I^2 [95% CI]
Extremely large VLDL	0.00 [0.00, 86.90]	27.42 [0.00, 97.20]
Very large VLDL	0.00 [0.00, 95.70]	0.00 [0.00, 93.81]
Large VLDL	6.66[0.00, 99.24]	0.00 [0.00, 87.79]
Medium VLDL	6.49 [0.00, 98.82]	0.00 [0.00, 89.13]
Small VLDL	0.00 [0.00, 98.07]	0.00 [0.00, 90.01]
Very small VLDL	14.71 [0.00, 96.29]	39.88 [0.00, 95.21]
IDL	24.11 [0.00, 96.01]	25.98 [0.00, 93.71]
Large LDL	20.75 [0.00, 95.25]	42.49 [0.00, 95.21]
Medium LDL	23.79 0.00, 93.93]	5.35 [0.00, 93.32]
Small LDL	20.62 [1.00, 94.51]	40.18 [0.00, 93.15]
Very large HDL	31.05 [0.92, 99.00]	0.00 [0.00, 94.44]
Large HDL	70.53 [24.79, 99.37]	33.87 [0.00, 99.20]
Medium HDL	0.00 [0.78, 94.99]	26.91 [0.00, 93.85]
Small HDL	38.94 [0.94, 95.86]	14.13 [0.00, 90.83]
Total TG	15.58 [0.00, 97.90]	9.51 [0.00, 93.64]

Estimates are heterogeneity statistics I^2 (%) and 95% confidence intervals (CI)

I^2 adjusted for; age, sex, body mass index, smoking, systolic blood pressure, type 2 diabetes.

CHD = Coronary heart disease; VLDL = Very-low density lipoprotein; IDL = Intermediate-density lipoprotein; LDL = Low-density lipoprotein; HDL = High-density lipoprotein.

Supplementary table 2 Effect estimates for NMR measured and clinical chemistry measured lipids with CHD

Lipid measure method	CHD OR	LB	UB
**NMR measured TG**	1.19	1.10	1.28
**Clinical chemistry measured TG**	1.14	1.07	1.21
**NMR measured total cholesterol**	1.10	1.02	1.19
**Clinical chemistry measured total cholesterol**	1.22	1.14	1.31

Estimates are odds ratios and 95% confidence intervals (CI)
OR adjusted for; age, sex, body mass index, smoking, systolic blood pressure, type 2 diabetes.
TG = triglycerides

CHD = coronary heart disease

OR = odds ratio

LB = lower band

UB = upper band

Supplementary figure 1 Evaluate the association of NMR measured total and 14 triglyceride sub-fraction with CHD independent of HDL-C and LDL-C

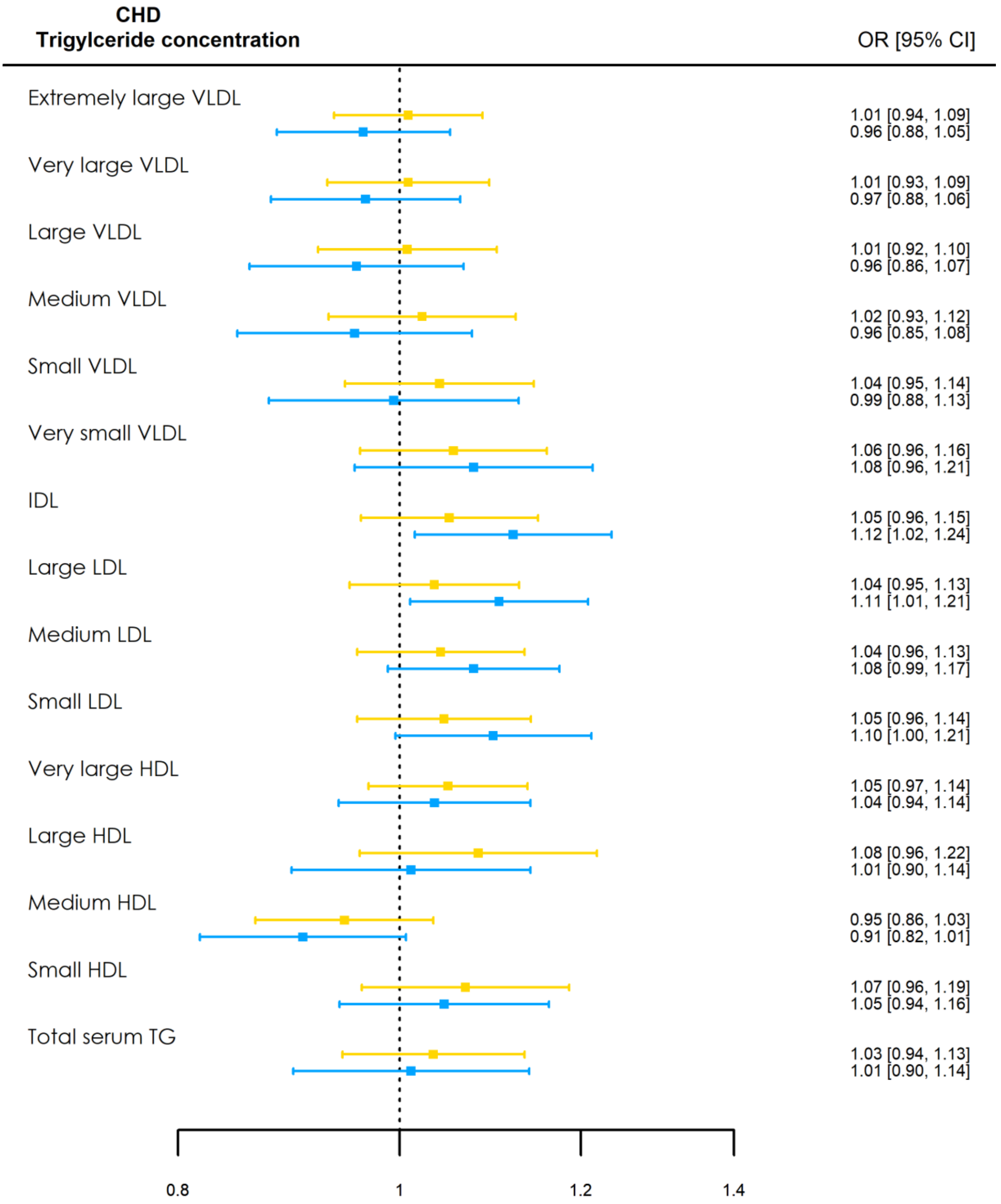

N.B. Effect estimates are presented as odds ratios (OR) with 95% confidence intervals (CI) per 1 standard deviation increase in the analyte for CHD. Models are adjusted for; age, sex, smoking status, BMI, systolic blood pressure, type 2 diabetes and; HDL-C and LDL-C (denoted by yellow bar), ApoA1 and ApoB (denoted by blue bar).

Supplementary figure 2 NMR measured total and 14 triglyceride sub-fraction associations with CHD

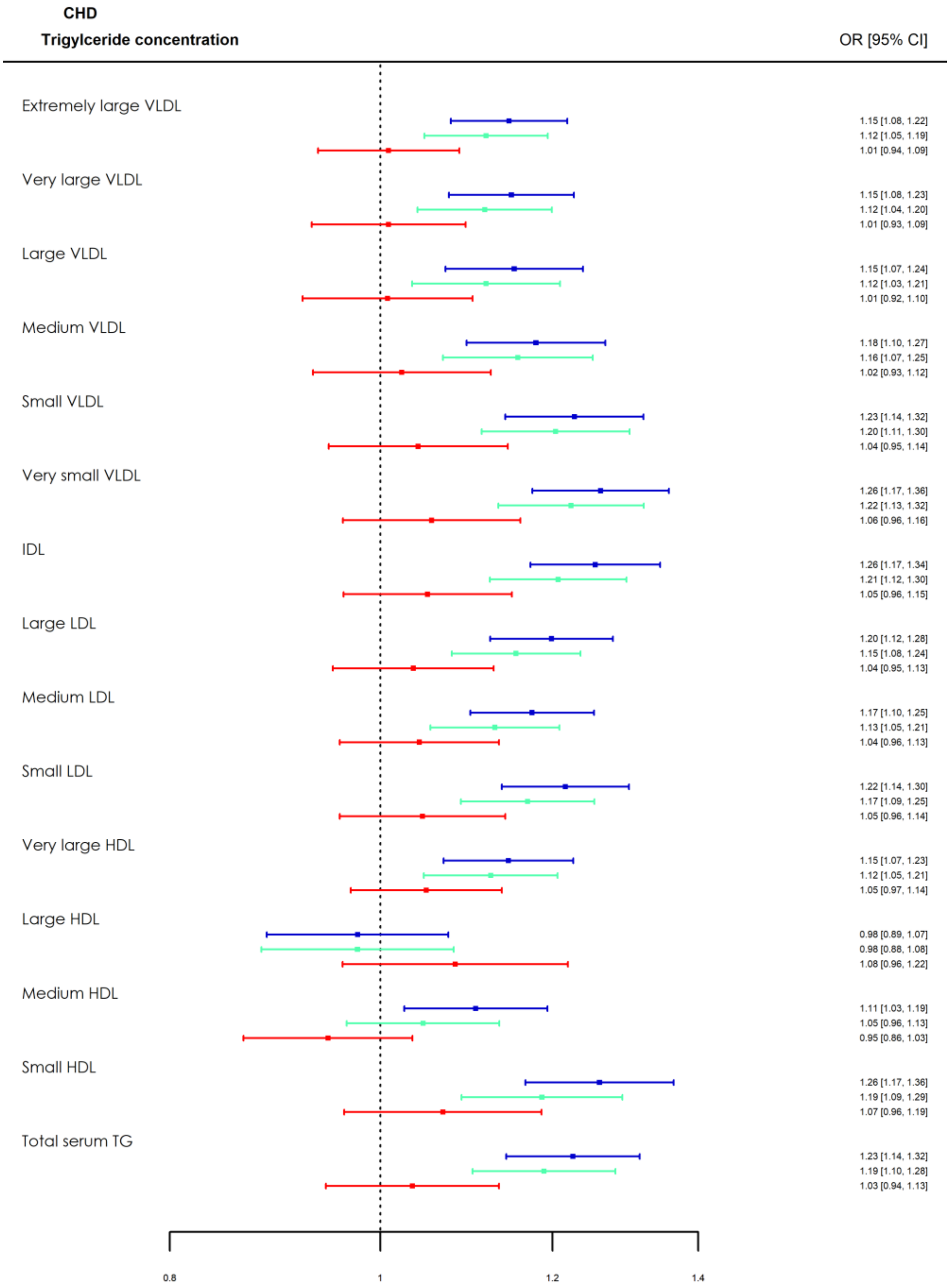

N.B. Effect estimates are presented as odds ratios (OR) with 95% confidence intervals (CI) per 1 standard deviation increase in the analyte for CHD. Models are adjusted for; age and sex (model 1 denoted by blue bar), model 1 with additional correction for smoking status, BMI, systolic blood pressure and type 2 diabetes (model 2 denoted by green bar). Model 2 with additional correction for HDL-C and LDL-C (model 3 denoted by red bar)

Supplementary figure 2 Total and 14 triglyceride sub-fraction measured in the fasting state and non-fasting state associations with CHD and stroke

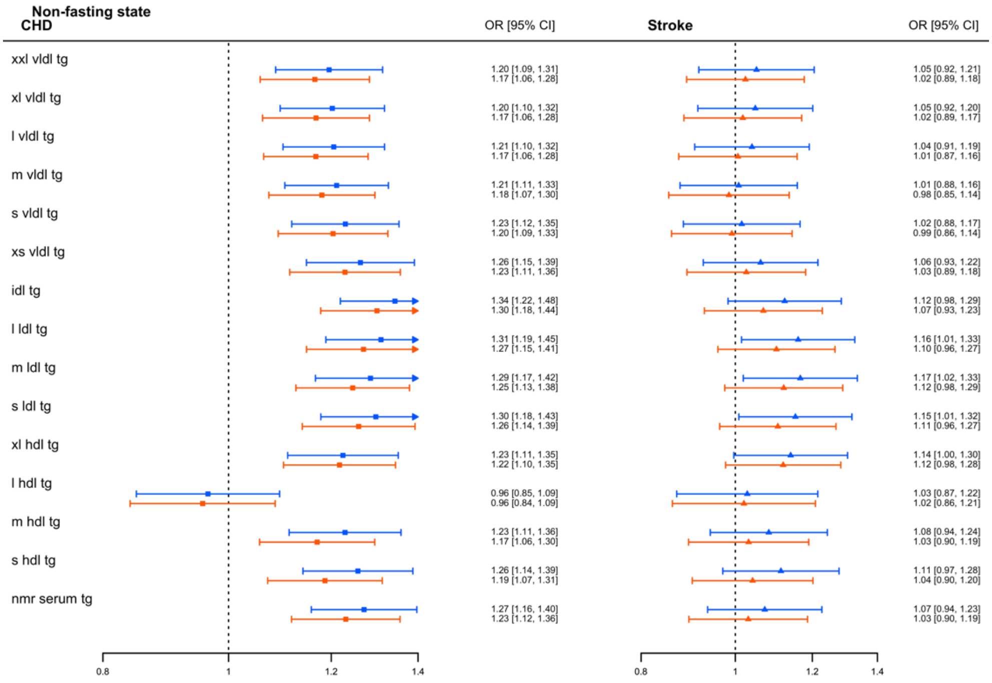

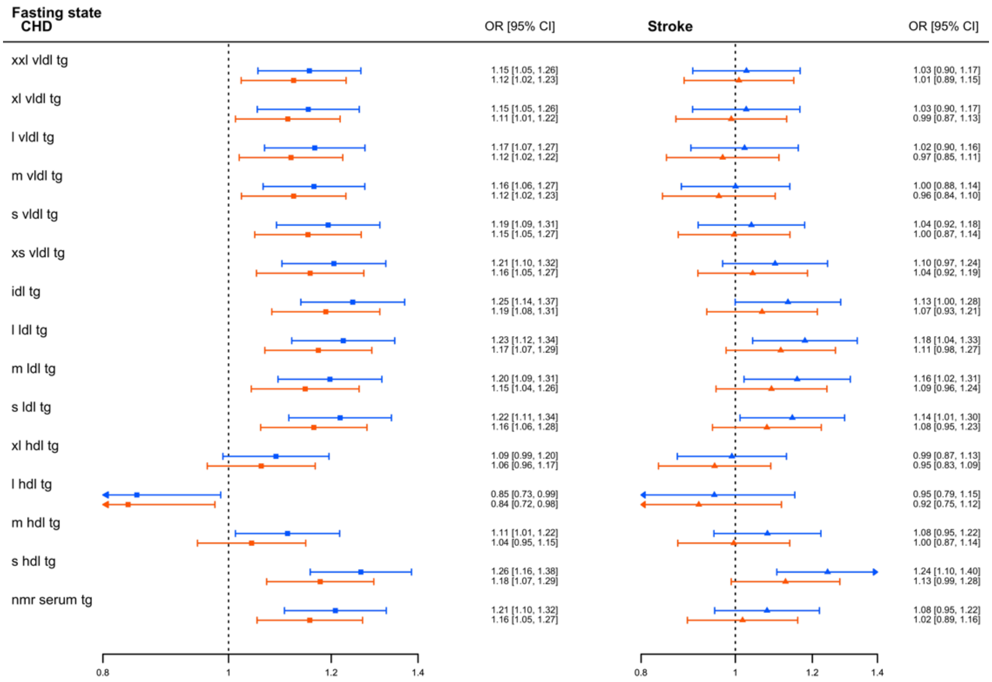

N.B. TG sub-fraction measures in; top: non-fasting state, bottom: fasting state. Effect estimates are presented as odds ratios (OR) with 95% confidence intervals (CI) per 1 standard deviation increase in the analyte for CHD and stroke. Models are adjusted for; age and sex (model 1 denoted by blue bar), model 1 with additional correction for smoking status, BMI, systolic blood pressure and type 2 diabetes (model 2 denoted by orange bar.
